# Assessing Child Maltreatment in Children Born to Mothers Who Used Methamphetamine during Pregnancy at Siriraj Hospital, Bangkok, Thailand: A Pilot Study

**DOI:** 10.1155/2014/406208

**Published:** 2014-11-18

**Authors:** Nontima Patcharoros, Sudsabuy Chulakadabba, Nattawadee Na Manorom, Vitharon Boon-yasidhi

**Affiliations:** ^1^Department of Psychiatry, Faculty of Medicine Siriraj Hospital, Mahidol University, Bangkok 10700, Thailand; ^2^Department of Pediatrics, Faculty of Medicine Siriraj Hospital, Mahidol University, Bangkok 10700, Thailand

## Abstract

Studies on maltreatment of children born to methamphetamine abusing mothers are lacking. This cross-sectional study examined child maltreatment among children born at Siriraj Hospital, Bangkok, Thailand, to mothers who used methamphetamines during pregnancy. During the study period between July 2011 and January 2012, 34 caretakers of these children were interviewed using the ISPCAN Child Abuse Screening Tool-Parent Version (ICAST-P) to assess their disciplinary actions. The associations between child's and caretaker's characteristics and child maltreatment behaviors were analyzed. More than 90% of caretakers were female with age ranging from 18 to 35 years and about 60% were biological mothers. The children's age ranged from 1 to 9 years. Disciplinary acts and child rearing practices that were considered to be child maltreatment behaviors were reported as follows: psychological discipline 82.4%, physical discipline 79.4%, and neglect 29.4%. No associations between the child's or the caretaker's characteristics and child maltreatment behaviors were found. In conclusion, child maltreatment behaviors were frequent in caretakers of children born to mothers who used methamphetamine during pregnancy. Supervision on child rearing and careful monitoring are needed for this population.

## 1. Introduction

Methamphetamine abuse remains a concerning problem in a number of regions worldwide including Thailand [1]. The increasing use of methamphetamine in young men and women leads to a number of social and health problems including risk-taking behaviors and unplanned pregnancy. The use of this substance during pregnancy has been noted to be associated with both maternal and neonatal morbidities [2]. As methamphetamine can cause a range of physical and mental health problems, including aggression, cognitive deficits, anxiety, depression, and psychosis [3], methamphetamine use during pregnancy and after delivery could impair child rearing function in the mothers. It was demonstrated in a matched-cohort study from Australia that infants of mothers who were abusing drugs, including amphetamine, were more likely to be abused and enter foster care [4]. A cross-sectional study in Thailand examining the health impact on neonates born to mothers who used methamphetamine during pregnancy indicated that 96% of the mothers had inadequate prenatal care and 39% were unwilling to take their children [5]. These data suggest that children born to mothers who used methamphetamine during pregnancy are at risk of being maltreated. However, there have been no systemic studies examining child maltreatment in this population.

Starting in 2002, a multidisciplinary team at Siriraj Hospital, one of the teaching hospitals in Bangkok, Thailand, developed a program of risk assessment and prevention of child maltreatment among infants born to mothers who used methamphetamine during pregnancy. Delivering mothers with a history of methamphetamine use or positive urine methamphetamine test were evaluated by psychiatrists and social workers. Those who were determined at risk of child maltreatment were evaluated by a child protection team to determine appropriate disposition. Children who were determined not necessary to be placed in child protection facilities were discharged to families and scheduled to come to the clinic for further assessment of the risk of maltreatment and continued support. These children were discharged to their biological mothers or to relatives who substituted for the mothers who continued using methamphetamine or were incapable of raising their own children. The present study was aimed at evaluating child maltreatment behaviors among caretakers of the children who were discharged to families by using a standard instrument and exploring factors related to maltreatment behaviors.

## 2. Material and Method

This was a cross-sectional study conducted at Siriraj Hospital during July 2011 and January 2012. The study subjects were children born at Siriraj Hospital between the years 2002 and 2011 to mothers who used methamphetamine during pregnancy, who were discharged to families and were being followed for assessment of the risk of maltreatment and continued support. The follow-up visits were scheduled every 3-4 months. Of the total of approximately 100 children who were being followed, 34 children came to the clinic visits during the study period and their caretakers were invited to participate. Exclusion criteria included caretakers who declined or were unable to participate in the interview process. Consenting caretakers were interviewed using the ISPCAN Child Abuse Screening Tool-Parent Version (ICAST-P) and the caretaker's and child's demographic and clinical data questionnaire developed by the investigators. Participants were interviewed only one time. The study was approved by the Siriraj Hospital Ethic Committee (COA number SI 316/2011).

### 2.1. The Instruments

The ISPCAN Child Abuse Screening Tool-Parent Version (ICAST-P) is a standard instrument for screening of child abuse developed by the International Society for the Prevention of Child Abuse and Neglect (ISPCAN). The ICASP-P was developed as a survey of child disciplinary practices that are considered child maltreatment [6]. The child disciplinary acts being surveyed are not the entire range of disciplinary behaviors but behaviors thought to be common and important by experts from 40 different countries. It has been validated in studies involving 697 parent respondents in 6 countries worldwide [6]. The instrument consists of 45 questions: 7 questions on general information and 38 questions on disciplinary acts, omission in care, and sexual abuse, which have occurred in the past year. Caretakers are asked whether they have ever done any of the listed specific disciplinary acts to their child over the past year and the frequency of those acts. Disciplinary practices are classified into nonviolent discipline, moderate physical discipline, severe physical discipline, neglect, and sexual abuse. In this study, caretaker's reports of moderate and severe physical discipline, psychological discipline, neglect, and sexual abuse were considered maltreatment behaviors.

The caretaker's and child's demographic and clinical data questionnaire is a semistructured questionnaire developed by the investigators. It consists of questions on caretaker's characteristics, such as age, educational level, occupation, history of being abused as a child, and history of substance abuse, and child's characteristics including physical disease, disability, developmental delay, and problem behaviors.

### 2.2. Statistical Analysis

The characteristics of the sample and child maltreatment behaviors were analyzed using descriptive statistics. The associations between caretaker's characteristics and each type of child maltreatment behaviors and between child's characteristic and each type of child maltreatment behaviors were analyzed using logistic regression analysis. The caretaker's variables included age, gender, relationship with child, history of being abused as a child, and history of substance abuse. The child's variables included age, gender, birth order, having developmental delay, and having problem behavior. All analyses were conducted using SPSS Statistics 16 (IBM SPSS, Chicago, IL).

## 3. Results

### 3.1. Caretaker's and Child's Demographic Data

Thirty-four caretakers were invited and all consented to interview during the study period. More than 90% of caretakers were female and about 60% were the biological mothers. History of substance abuse was reported in more than 60%, while history of being abused as a child and history of psychiatric treatment were reported by only a few caretakers. The caretaker's characteristics are shown in Table 1.

Of the 34 recruited children, 60% were female. The children's ages ranged from 1 month to 9 years with the median of 2 years. About half of the children were the first child born to the mothers. Developmental delay and problem behaviors were reported in 17% and 29% of the children, respectively. The children's characteristics are shown in Table 2.

### 3.2. Child Maltreatment Behaviors

Disciplinary practices that were considered child maltreatment behaviors are shown in Table 3. Eighty-two percent of caretakers reported using psychological discipline of their child. Moderate or severe physical discipline and neglect were reported in 79.4% and 29.4%, respectively, while sexual abuse acts were not reported.

The two most frequent psychological discipline acts reported were threatened to abandon (58.8%) and shouted (55.9%), followed by threatened to invoke spirits (38.2%) and threatened to send away (38.2%). The most frequent severe physical discipline acts included shook child under 2 years (26.1%) and kicked (11.8%). The most frequent moderate physical discipline acts included spanked (58.8%), shook child over 2 years (36.4%), hit elsewhere with objects (20.6%), and pinched (20.6%).

### 3.3. The Relationship of Caretaker's and Child's Characteristics and Child Maltreatment Behaviors

Caretaker's and child's characteristics were analyzed to assess the association with each type of child maltreatment behaviors using logistic regression analysis. It was found that caretaker's age, being the biological mother, being unemployed, educational level, having history of substance abuse, and family income were not associated with child maltreatment behaviors. Likewise, child's age, gender, and birth order were not associated with child maltreatment behaviors (data not shown). Other characteristics could not be assessed due to the too small number of variables in each category.

## 4. Discussion

This study used the ICAST-P to examine child maltreatment behaviors in caretakers of children, born to mothers who used methamphetamine during pregnancy, who were discharged to families and were being followed at Siriraj Hospital. These children were among those who were determined at risk of child abuse but were not necessary to be placed in child protection facilities. We found disciplinary acts considered to be physical abuse and psychological abuse were reported in approximately 80% and behaviors considered to be neglect were reported in approximately 30% of the caretakers of these children. These numbers reflect concerning levels of inappropriate disciplinary acts and child rearing behaviors in caretakers of this group of children.

These high percentages of child maltreatment behaviors reported in this study might be due to the fact that any single disciplinary act of the ICAST-P was counted regardless of frequency of the acts. Therefore these reported disciplinary acts could not be substantiated as child maltreatment. The ICAST-P is an instrument for the survey of child disciplinary practices that are considered child maltreatment and not a diagnostic tool for child maltreatment. Nonetheless, the high occurrence of such behaviors is concerning and reflects the need for continued supervision and monitoring in this population. We did not find sexual abusive behaviors reported in any children. This might be due to the fact that most caretakers in this study were female and most of the children were still very young. This, however, does not imply that these children are not at risk of sexual abuse.

In this study, only about 60% of the children were taken care of by the biological mother and 40% were cared after by other relatives. We did not find differences in child maltreatment behaviors among caretakers who were biological mothers and those who were other relatives. This suggests that, although it was determined that the families of both groups (the biological mothers and other relatives) were safe for the children to be discharged to, child maltreatment behaviors were found to be high and not different among the groups.

We also failed to find an association of any other caretaker's and child's factors with child maltreatment behaviors. While it has been found in other studies that child maltreatment was associated with parental substance abuse history [7, 8], we did not find an association between child maltreatment behaviors and substance abuse history in the caretakers by logistic regression analysis. This might be due to the small sample size in this pilot study.

There are some limitations in this study. Firstly, the method of assessment by interviewing caretakers might be subject to recall bias. The number of reported behaviors could be either lower or higher than what actually occurred. Moreover, the caretaker's report might not be truthful or accurate. Secondly, we did not have data on children born to methamphetamine using mothers who did not come for follow-up. Thirdly, there is no normative data for the ICAST-P as it is a survey tool, and there was no control group for comparison in this study. Finally, this study had a small sample size; no conclusion regarding factors associated with child maltreatment can be drawn from this pilot study.

Despite these limitations, this pilot study provides interesting findings that child maltreatment behaviors can be found at high percentages in caretakers of children born to mothers who used methamphetamine during pregnancy. This highlights the importance of careful evaluation, supervision, and support for this population, including risk assessment for child maltreatment and pursuing child protection interventions when necessary.

## 5. Conclusion

The results of the present study reveal that disciplinary acts considered to be child maltreatment among caretakers of children born to mothers who used methamphetamine during pregnancy were found to be high, particularly physical discipline and psychological discipline. This suggests that child rearing guidance and careful monitoring are needed for these families.

## Figures and Tables

**Table 1:** Caretaker's characteristics.

Characteristics	
Age: median, range (year)	30, 18–64
Gender, female: *n* (%)	32 (94.1)
Relationship to the child: *n* (%)	
Mother	20 (58.8)
Other relatives	14 (41.2)
Educational level: *n* (%)	
Lower than primary education	18 (52.9)
Higher than primary education	16 (47.1)
Unemployed: *n* (%)	18 (52.9)
History of being abused as a child: *n* (%)	1 (2.9)
History of psychiatric treatment: *n* (%)	3 (8.8)
History of substance abuse: *n* (%)	22 (64.7)
Family income: *n* (%)	
Below 10,000 bahts/month	24 (70.6)
Over 10,000 bahts/month	10 (29.4)

**Table 2:** Child's characteristics.

Characteristics	
Age: median, range (year)	2, 0–9
Gender, female: *n* (%)	21 (61.8)
Birth order as first child: *n* (%)	15 (44.1)
Having developmental delay: *n* (%)	6 (17.6)
Having problem behaviors: *n* (%)	10 (29.4)

**Table 3:** Frequency and percentage of child maltreatment behaviors.

Child maltreatment behaviors	*N*	Percentage
Physical discipline (total)	**27**	**79.4**
Severe physical discipline		
Shook child under 2 years^*^	6	26.1
Kicked	4	11.8
Beat up	3	8.8
Threaten with knife or gun	1	2.9
Moderate physical discipline		
Spanked	20	58.8
Hit on buttocks with object	8	23.5
Hit elsewhere with object	7	20.6
Pinched	7	20.6
Pulled hair	5	14.7
Shook child over 2 years^*^	4	36.4
Twisted ear	4	11.8
Knuckled back of head	4	11.8
Chili pepper in mouth	3	8.8
Slapped back of head	1	2.9
Psychological discipline (total)	**28**	**82.4**
Threatened to abandon	20	58.8
Shouted	19	55.9
Threatened to invoke spirits	13	38.2
Threatened to send away	13	38.2
Refused to speak	10	29.4
Insulted	6	17.6
Public humiliation	3	8.8
Lock out of house	1	2.9
Lock in dark room	1	2.9
Neglect (total)	**10**	**29.4**
Hurt, inadequate supervision	7	20.6
Inadequate food, liquid	2	5.9
Unmet medical need	1	2.9
Sexual abuse (total)	** 0**	**0**

^*^ *n* = 34 except for shook child under 2 years (*n* = 23) and over 2 years (*n* = 11).
